# Necrotrophic growth of periodontopathogens is a novel virulence factor in oral biofilms

**DOI:** 10.1038/s41598-017-01239-9

**Published:** 2017-04-24

**Authors:** Esteban Rodriguez Herrero, Nico Boon, Martine Pauwels, Kristel Bernaerts, Vera Slomka, Marc Quirynen, Wim Teughels

**Affiliations:** 1 Department of Oral Health Sciences, KU Leuven, Kapucijnenvoer 33, 3000 Leuven, Belgium; 2 Center for Microbial Ecology and Technology (CMET), Ghent University, Coupure links 653, 9000 Gent, Belgium; 3 Bio- and Chemical Systems Technology, Reactor Engineering and Safety, Department of Chemical Engineering, KU Leuven (University of Leuven), Leuven Chem&Tech, Celestijnenlaan 200F (bus 2424), 3001 Leuven, Belgium; 4 Dentistry, University Hospitals Leuven, Kapucijnenvoer 33, 3000 Leuven, Belgium

## Abstract

The oral use of antimicrobial agents embedded in toothpastes and mouth rinses results in an oral microbial massacre with high amounts of dead bacteria in close proximity to few surviving bacteria. It was hypothesized that this provides the surviving pathogenic bacteria a large amount of dead microbial biomass as a nutritional source for growth (necrotrophy). This study demonstrated the necrotrophic growth of periodontal pathogens in the presence of different dead oral species. In addition, the presence of dead bacteria resulted in an outgrowth of several periodontal pathogens in complex multi-species biofilms. Additionally, upon contact with dead oral bacteria, virulence genes of *P*. *intermedia* and *P*. *gingivalis* were up-regulated (necrovirulence). This resulted in a more pronounced epithelial cytotoxicity (necrotoxicity). These findings indicate that presence of dead bacteria induce necrotrophy, necrovirulence and necrotoxicity in several oral pathogens.

## Introduction

Dental plaque is a complex poly-microbial biofilm in which more than 700 different microbial species can reside^1^. These microbial communities develop on all hard surfaces and soft tissues in the oral cavity and are embedded in extracellular polymeric matrix. The biofilm structure confers new properties to planktonic bacteria in terms of adhesion, communication, protection against the environment, the host’s immune response and antimicrobials^2^. Bacterial-host-environmental interactions determine the structure, composition and metabolic activity of these biofilms. All of these play an important role in the development of some of the most common diseases of mankind: periodontal diseases and caries. Periodontitis is an infectious disease resulting in a destruction of the tooth or implant supporting tissues. A relationship exists between periodontitis as a chronic infection and several systemic diseases^3^.

Prevention of caries and plaque-induced periodontal diseases consists of regular disruption and removal of accumulating oral biofilms, primarily from teeth^4^. Mechanical plaque removal by means of tooth and interdental brushing remains the foundation stone of prevention^5^. However, individuals are often unable to accomplish this due to, among others, motivational issues and technical difficulties^5^. Consequently, they leave partially disrupted oral biofilms on teeth and soft tissues for days, weeks, up to months.

Adjunctive chemical approaches to control oral biofilms are widespread under the form of toothpastes and mouth rinses, which often contain antiseptic ingredients such as chlorhexidine (CHX), cetylpyridinium chloride (CPC) or triclosan (TC)^6^. They are considered as adjuncts and normally do not substitute mechanical plaque control by means of brushing^6^. Moreover, they will not remove oral biofilms but are used to chemically kill oral bacteria. It is well known that bacteria have developed ingenious ways to survive killing by antimicrobials. Especially, the properties associated with a biofilm lifestyle protect them. There is ample of data showing that the antiseptics in toothpastes or mouth rinses only kill bacteria in the outer layer of the biofilm^7^. Although a 99.9% killing efficiency (2–3 Log reductions) can be reached 30 minutes after a single application of e.g. CHX or stannous fluoride, still 10^5^ bacteria/mL will survive^8^. Consequently, the daily use of antimicrobial oral hygiene products containing antiseptics, results in a daily oral microbial massacre with high amounts of dead bacteria in close proximity to many surviving bacteria. Although these survivors can recover quickly from an antiseptic assault^9^, the fate of the bacterial casualties and their impact on survivors is currently unknown and has never been explored.

Recently it was shown that certain bacterial species can use dead microbial cells as a nutritional source for their own survival. *Legionella pneumophila*, for example, can use the remaining dead bacteria after heat treatment as a nutritional source to persist in water piping^10, 11^. This feature has been termed “bacterial necrotrophy”^11^. It is also known that starving *Bacillus subtilis* cells can produce toxins to kill sibling bacteria for nutrition^12^. The nutrients released by this behaviour from the killed siblings are being used to feed the surviving bacteria under conditions of nutritional stress^13^. By this, *B*. *subtilis* can prolong its survival and delay or avoid going into sporulation, which is a time and energy consuming process. This behaviour has been termed “cannibalism”^14^.

The widespread and daily use of antiseptic mouth rinses and toothpastes together with the inability for most people to accomplish a complete dental plaque removal makes that bacterial necrotrophy might be relevant for the oral ecosystem. Therefore, it is crucial to clarify if and how oral microbial ecosystems respond to sudden increases in dead bacteria.

The aim of this study was to determine if periodontopathogens can use dead bacteria as a nutritional substrate and to determine the impact of dead bacteria on bacterial virulence.

## Results

### Effect of dead bacteria on bacterial growth

In order to evaluate the necrotrophic behaviour of *P*. *intermedia*, a culture of *P*. *intermedia* was supplemented with dead *P*. *intermedia*. As shown in Table 1, after 24 hours, the total number of *P*. *intermedia* (CFU/mL) was significantly increased in the culture supplemented with dead *P*. *intermedia* when compared to a not-supplemented culture. This increase was dose dependent and resulted in a maximum supplemental outgrowth of *P*. *intermedia* of up to 1.46 (±0.01) Log_10_ CFU/mL in the presence of dead *P*. *intermedia*.

**Table 1:** Effect of dead bacteria on bacterial growth of periodontal pathogens.

Living Bacteria	Dead bacteria	Final Log_10_ (CFU/mL)
*P*. *intermedia*	10^9^ *P*. *intermedia*	9.48 ± 0.03*
	10^8^ *P*. *intermedia*	8.68 ± 0.04*
	10^7^ *P*. *intermedia*	8.13 ± 0.07*
	Control (without dead *P*. *intermedia*)	8.02 ± 0.03*
	10^9^ *P*. *gingivalis*	9.84 ± 0.18*
	10^8^ *P*. *gingivalis*	9.48 ± 0.13*
	10^7^ *P*. *gingivalis*	9.22 ± 0.01*
	Control (without dead *P*. *gingivalis*)	8.90 ± 0.04*
	10^9^ *S*. *oralis*	9.37 ± 0.02*
	10^8^ *S*. *oralis*	9.28 ± 0.09*
	10^7^ *S*. *oralis*	9.24 ± 0.07*
	Control (without dead *S*. *oralis*)	9.13 ± 0.09*
	10^9^ *F*. *nucleatum*	9.46 ± 0.04*
	10^8^ *F*. *nucleatum*	9.43 ± 0.02*
	10^7^ *F*. *nucleatum*	9.37 ± 0.01*
	Control (without dead *F*. *nucleatum)*	9.34 ± 0.02*
	10^9^ *S*. *gordonii*	9.43 ± 0.05*
	10^8^ *S*. *gordonii*	9.36 ± 0.04*
	10^7^ *S*. *gordonii*	9.35 ± 0.06*
	Control (without dead *S*. *gordonii*)	9.32 ± 0.04*
	10^9^ *P. gingivalis* + 1 in 10 BHI	6.42 ± 0.10*
	Control 1 in 10 BHI (without dead *P*. *gingivalis*)	4.65 ± 0.62*
*P*. *gingivalis*	10^9^ *P*. *gingivalis*	9.10 ± 0.08*
	10^8^ *P*. *gingivalis*	9.06 ± 0.10*
	10^7^ *P*. *gingivalis*	8.94 ± 0.05*
	Control (without dead *P*. *gingivalis)*	8.78 ± 0.04*
*F*. *nucleatum*	10^9^ *F*. *nucleatum*	8.59 ± 0.03*
	10^8^ *F*. *nucleatum*	8.48 ± 0.06*
	10^7^ *F*. *nucleatum*	8.45 ± 0.08*
	Control (without dead *F*. *nucleatum)*	8.38 ± 0.06*

Data represent the final cell concentration of living periodontal pathogens (Log_10_ CFU/mL)after 24 anaerobic incubation starting from 7.7 Log_10_ CFU/mL and in presence of different concentrations of dead bacteria (mean ± standard deviation, n = 3). Control contains only living bacteria without addition of dead bacteria. *Statistically significant difference of periodontal pathogens concentration in presence of dead bacteria respect to the control (p < 0.05).

The necrotrophic behaviour of *P*. *intermedia* was however depending on the dead species. Supplementing a *P*. *intermedia* culture with either dead *P*. *gingivalis*, *F*. *nucleatum*, *S*. *oralis* or *S*. *gordonii* resulted in a significant increase of *P*. *intermedia* growth (Table 1). However, dead *S*. *gordonii* only increased the growth of *P*. *intermedia* in its highest concentration whereas dead *P*. *gingivalis* and dead *F*. *nucleatum* significantly increased the growth of *P*. *intermedia* in all used concentrations with up to 0.94 (±0.26) Log_10_ CFU/mL. Also for these dead species, a dose dependent effect was observed.

Necrotrophic behaviour was also observed for *F*. *nucleatum* and *P*. *gingivalis* in the presence of dead *F*. *nucleatum* and dead *P*. *gingivalis* respectively (Table 1). However, the magnitude of the growth increase induced by supplementing these species with dead bacteria was clearly lower than for *P*. *intermedia*. All concentrations (10^9^, 10^8^ and 10^7^ CFU/mL) of dead *P*. *gingivalis* and dead *F*. *nucleatum* could increase the growth of *P*. *gingivalis* (Table 1).

Since the above described experiments were performed in a nutrient rich medium, it could be hypothesized that the effect of supplementing dead bacteria could be more pronounced in nutrient limited media. This was examined by supplementing *P*. *intermedia* with dead *P*. *gingivalis* in a nutrient limited medium BHI diluted 1/10 or 1/100 in PBS. After 24 hours of incubation, the concentration of *P*. *intermedia* in 1/10 BHI was reduced to 4.65 (±0.77) Log_10_ CFU/mL, indicating starvation of *P*. *intermedia*. However, if *P*. *intermedia* in 1/10 BHI was supplemented with dead *P*. *gingivalis*, the number of surviving bacteria was 6.42 (±0.13) Log_10_ CFU/mL (Table 1). The latter was significantly (p < 0.05) more than in the not supplemented condition and points towards a higher survival rate when dead *P*. *gingivalis* was present. When the culture media was further depleted from nutrients (1/100 BHI), no surviving bacteria could be detected for both conditions (data not shown).

### The fate of dead bacteria

In order to further substantiate the necrotrophic behaviour, the DNA of dead bacteria was monitored over time by qPCR and correlated to the number of consumed cells.

As shown in Table 2, the qPCR data confirmed the dose dependent culturing data showing that when *P*. *intermedia* is supplemented with dead *P*. *gingivalis* or dead *S*. *oralis*, its growth over 24 hours is increased with up to 1.07 (±0.12) Log_10_ Geq/mL and with up to 0.32 (±0.17) Log_10_ Geq/mL respectively (p < 0.05). Additionally, the qPCR data showed that the DNA concentrations of dead *P*. *gingivalis* and *S*. *oralis* decreased significantly after 24 hours of incubation with living *P*. *intermedia*. No decrease was seen in control solutions containing only dead *P*. *gingivalis* and *S*. *oralis* without living *P*. *intermedia*. The DNA concentrations of dead *P*. *gingivalis* decreased for all concentrations of dead bacteria with up to 0.77 Log_10_ Geq/mL whereas the DNA concentrations of dead *S*. *oralis*, decreased for all concentrations of dead bacteria with up to 1.99 Log_10_ Geq/mL.

**Table 2:** Necrotrophic activity of *P*. *intermedia* and DNA concentrations of dead *P*. *gingivalis* and dead *S*. *oralis* (Log (MO/mL) analyzed by qPCR (mean ± standard deviation, n = 3).

A			
Living Bacteria	Dead bacteria	Log (CFU/mL) (Vitality qPCR)	Log (CFU/mL) (Standard qPCR)
*P*. *intermedia*	10^9^ *P*. *gingivalis*	10.43 ± 0.04*	10.76 ± 0.07*
	10^8^ *P*. *gingivalis*	10.10 ± 0.10*	10.65 ± 0.03*
	10^7^ *P*. *gingivalis*	9.63 ± 0.20	9.72 ± 0.13
	Control	9.36 ± 0.06	9.47 ± 0.10
*P*. *intermedia*	10^9^ *S*. *oralis*	10.20 ± 0.09*	10.20 ± 0.11*
	10^8^ *S*. *oralis*	9.78 ± 0.08	10.12 ± 0.08*
	10^7^ *S*. *oralis*	9.71 ± 0.08	9.73 ± 0.11
	Control	9.88 ± 0.05	9.68 ± 0.04
**B**			
**Living Bacteria**	**Dead bacteria**	**Initial Log** (**[DNA dead bacteria]/mL**)	**Final Log** (**[DNA dead bacteria]/mL**)
*P*. *intermedia*	10^9^ *P*. *gingivalis*	8.29 ± 0.06	7.89 ± 0.11^#^
	10^8^ *P*. *gingivalis*	7.26 ± 0.14	6.48 ± 0.24^#^
	10^7^ *P*. *gingivalis*	6.27 ± 0.07	5.89 ± 0.04^#^
Control (without *Pi*)	10^9^ *P*. *gingivalis*	8.31 ± 0.06	8.15 ± 0.13*
*P*. *intermedia*	10^9^ *S*. *oralis*	7.32 ± 0.22	5.87 ± 0.18^#^
	10^8^ *S*. *oralis*	6.92 ± 0.03	6.37 ± 0.35*
	10^7^ *S*. *oralis*	5.66 ± 0.33	3.67 ± 0.19*
Control (without *Pi*)	10^9^ *S*. *oralis*	7.73 ± 0.44	7.55 ± 0.50*

(A) Necrotrophic activity of *P*. *intermedia* in presence of dead *P*. *gingivalis* and dead *S*. *oralis* analyzed by standard and vitality qPCR. Control contains only living *P. intermedia*. *Designates a statistically significant outgrowth of *P*. *intermedia* respect to control (p < 0.05). (B) Initial and final concentrations of DNA from dead *P*. *gingivalis* and dead *S*. *oralis* during the necrotrophic activity of *P*. *intermedia* analyzed by standard qPCR. Control contains only DNA of dead bacteria. ^#^Designates a statistically significant decrease of DNA from dead bacteria respect to control (p < 0.05).

### Necrotrophic growth in a multispecies community

Necrotrophic activity in the presence of other bacteria was tested to check for competitive effects. An increase in the concentrations of periodontal pathogens was observed inside of the 14 species planktonic community by the presence of dead *P*. *intermedia* and *P*. *gingivalis* in BHI-2, saliva or serum (Table 3, Supplementary Table 1, Supplementary Table 2 and Supplementary Table 3). The presence of dead *P*. *intermedia* increased the growth of living *P*. *intermedia* in BHI-2, saliva and serum (p < 0.05). *F*. *nucleatum* and *A*. *actinomycetemcomitans* increased also significantly by the addition of dead *P*. *intermedia* in BHI-2. Only the presence of dead *P*. *intermedia* increased the concentrations of *P*. *gingivalis* and *F*. *nucleatum* in saliva. Moreover the presence of dead *P*. *gingivalis* increased significantly the growth of *P*. *intermedia*, *P*. *gingivalis* and *F*. *nucleatum* in BHI-2, of *P*. *gingivalis* and *F*. *nucleatum* in saliva, and of *P*. *intermedia*, *A*. *actinomycetemcomitans* and *P*. *gingivalis* in serum.

**Table 3:** Vitality qPCR (Log (MO/mL) of the different species (mean ± standard deviation, n = 3) in the 14 species community in presence of living and dead bacteria in BHI-2 after 24 hours incubation.

	Planktonic					Biofilm				
	BHI-2	+dead Pi	+Living Pi	+dead Pg	+Living Pg	BHI-2	+dead Pi	+Living Pi	+dead Pg	+Living Pg
*Aa*	7.27 ± 0.11	7.93 ± 0.10*	6.54 ± 0.07^#^	7.69 ± 0.05*	7.32 ± 0.08	6.88 ± 0.03	7.56 ± 0.10*	6.11 ± 0.11^#^	6.40 ± 0.11^#^	6.99 ± 0.15
*Pi*	7.15 ± 0.14	7.70 ± 0.11*	7.56 ± 0.07*	8.75 ± 0.06*	6.89 ± 0.04	8.16 ± 0.03	8.97 ± 0.19*	8.65 ± 0.04*	8.72 ± 0.06*	7.64 ± 0.07^#^
*Pg*	8.05 ± 0.09	8.15 ± 0.09	7.98 ± 0.05	8.83 ± 0.10*	10.56 ± 0.03*	7.81 ± 0.03	8.08 ± 0.11*	8.05 ± 0.05*	8.41 ± 0.06*	9.75 ± 0.13*
*Fn*	8.08 ± 0.05	9.26 ± 0.17*	8.89 ± 0.08*	10.08 ± 0.30*	9.08 ± 0.01*	8.93 ± 0.12	10.19 ± 0.13*	9.82 ± 0.18*	9.89 ± 0.11*	9.56 ± 0.14*
*A*. *naeslundii*	4.58 ± 0.09	3.28 ± 0.17^#^	4.98 ± 0.17	4.87 ± 0.15	4.52 ± 0.36	6.77 ± 0.05	3.94 ± 0.37^#^	5.95 ± 0.03^#^	6.82 ± 0.11	6.86 ± 0.16
*A*. *viscosus*	6.37 ± 0.15	2.95 ± 0.12^#^	5.75 ± 0.11^#^	6.27 ± 0.18	6.63 ± 0.17*	7.77 ± 0.07	4.85 ± 0.49^#^	7.00 ± 0.01^#^	7.67 ± 0.06	7.54 ± 0.12
*S*. *mutans*	7.01 ± 0.22	5.78 ± 0.12^#^	7.08 ± 0.21	7.27 ± 0.18*	6.09 ± 0.17^#^	8.09 ± 0.04	6.22 ± 0.30^#^	8.21 ± 0.10	7.78 ± 0.07^#^	7.82 ± 0.14
*S*. *sobrinus*	6.06 ± 0.15	5.66 ± 0.20^#^	6.07 ± 0.08	6.40 ± 0.11*	4.95 ± 0.21^#^	6.65 ± 0.03	6.94 ± 0.16	7.10 ± 0.13*	6.39 ± 0.08^#^	6.90 ± 0.25
*S*. *sanguinis*	7.78 ± 0.13	4.41 ± 0.15^#^	6.39 ± 0.05^#^	7.84 ± 0.05	6.47 ± 0.10^#^	8.21 ± 0.02	6.55 ± 0.37^#^	7.37 ± 0.07^#^	7.67 ± 0.07^#^	8.64 ± 0.14
*S*. *gordonii*	9.39 ± 0.16	8.67 ± 0.12^#^	7.28 ± 0.19^#^	9.44 ± 0.18	8.65 ± 0.06^#^	10.38 ± 0.05	9.17 ± 0.28^#^	9.43 ± 0.08^#^	10.06 ± 0.12^#^	10.33 ± 0.17
*S*. *oralis*	7.78 ± 0.07	3.93 ± 0.16^#^	5.61 ± 0.15^#^	7.35 ± 0.23	8.03 ± 0.05*	7.99 ± 0.01	5.60 ± 0.29^#^	6.58 ± 0.02^#^	7.64 ± 0.05^#^	8.25 ± 0.18
*S*. *salivarius*	5.98 ± 0.12	5.47 ± 0.20^#^	4.61 ± 0.20^#^	6.34 ± 0.23*	6.00 ± 0.19	7.39 ± 0.05	6.66 ± 0.35	6.02 ± 0.07^#^	6.76 ± 0.08^#^	6.90 ± 0.21
*V*. *parvula*	8.01 ± 0.07	8.65 ± 0.15*	8.51 ± 0.06*	8.26 ± 0.06*	9.01 ± 0.07*	10.33 ± 0.06	10.60 ± 0.08*	10.06 ± 0.10^#^	10.45 ± 0.07	10.16 ± 0.14
*S*. *mitis*	5.99 ± 0.15	2.80 ± 0.27^#^	4.81 ± 0.16^#^	6.16 ± 0.10*	5.19 ± 0.14^#^	6.77 ± 0.27	4.05 ± 0.21^#^	5.11 ± 0.06^#^	6.25 ± 0.03^#^	6.64 ± 0.08

BHI-2 condition refers to a 14 species community without the addition of dead bacteria. *Designates a statistically significant increase of the bacterial concentration with respect to BHI (p < 0.05). ^#^Designates a statistically significant decrease of the bacterial concentration in respect to BHI (p < 0.05).

In complex multi-species biofilms in BHI-2, the presence of dead *P*. *intermedia* resulted in an increased growth of *P*. *gingivalis*, *P*. *intermedia*, *A*. *actinomycetemcomitans* and *F*. *nucleatum* of up to 0.27 (±0.11), 0.82 (±0.16), 0.68 (±0.11) and 1.26 (±0.25) Log_10_ CFU/mL. Additionally, the presence of dead *P*. *intermedia* increased in serum only the growth of *P*. *intermedia* and *F*. *nucleatum* with up to 1.09 (±0.21) and 0.67 (±0.10) Log_10_ CFU/mL. In saliva the growth of *P*. *intermedia*, *P*. *gingivalis* and *F*. *nucleatum* was increased up to 0.32 (±0.03), 0.50 (±0.09) and 0.41(±0.07) Log_10_ CFU/mL.

Furthermore, the presence of dead *P*. *gingivalis* increased the growth of *P*. *gingivalis*, *P*. *intermedia* and *F*. *nucleatum* in complex multi-species biofilms up to 0.59 (±0.02), 0.56 (±0.09) and 0.97 (±0.23) Log_10_ CFU/mL in BHI, 0.47(±0.09), 0.79 (±0.08) and 1.36(±0.20) Log_10_ CFU/mL in serum and 0.16 (±0.08), 0.16 (±0.10) and 0.45 (±0.08) Log_10_ CFU/mL in saliva.

The effect of dead *P*. *intermedia* and dead *P*. *gingivalis* was also examined on the rest of bacterial species in the 14 species community (Table 3, Supplementary Table 1, Supplementary Table 2 and Supplementary Table 3). The presence of dead *P*. *intermedia* decreased the growth of *S*. *sanguinis*, *S*. *mitis*, *S*. *gordonii*, *S*. *oralis*, *A*. *viscosus*, *A*. *naeslundii*, *S*. *mutans* in BHI-2 and saliva (p < 0.05). Additionally, the presence of dead *P*. *gingivalis* decreased the concentrations of *S*. *sanguinis*, *S*. *salivarius*, *S*. *gordonii*, *S*. *mitis*, *S*. *oralis*, *S*. *mutans* and *S*. *sobrinus* in BHI-2 (p < 0.05) and of *S*. *gordonii*, *S*. *oralis*, *A*. *viscosus*, *A*. *naeslundii*, *S*. *mitis* in saliva (p < 0.05). A significant increase of *V*. *parvula* in BHI-2 was only reported by the presence of *P*. *intermedia*. Furthermore the presence of dead *P*. *gingivalis* in serum increased significantly the growth of *S*. *sanguinis*, *A*. *naeslundii*, *A*. *viscosus*, *S*. *gordonii*, *S*. *oralis*, *V*. *parvula*, *S*. *mutans* and *S*. *sobrinus* (p < 0.05).

### Virulence gene expression

**Figure 1:**
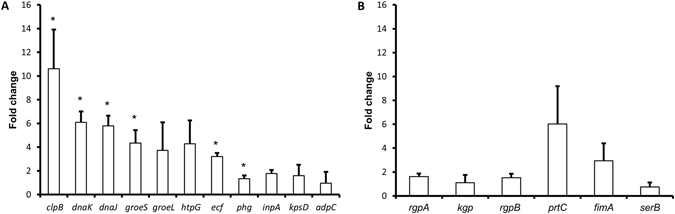
Expression of virulence genes from *P*. *intermedia* and *P*. *gingivalis* in presence of dead oral bacteria. (**A**) Relative fold change of virulence genes from *P*. *intermedia* after exposure to dead *P*. *gingivalis*. (**B**) Relative fold change of virulence genes from *P*. *gingivalis* after exposure to dead *P*. *intermedia*. *Designates a statistically significant up-regulation of virulence genes respect to house-keeping genes (p < 0.05).

Gene expression of *P*. *intermedia* and *P*. *gingivalis* was analysed after supplementing the cultures with dead *P*. *gingivalis* and dead *P*. *intermedia* respectively. After 24 hours of growth, the *P*. *intermedia* virulence genes *kpsD*, *phg*, *ecf*, *inpA* and *adpC* were 1.96 to 4.21 fold up-regulated when compared to the control culture (Fig. 1A). *P*. *intermedia* heat shock protein genes *htpG*, *dnaJ*, *dnaK*, *groeL*, *groeS*, *clpB*, were also up-regulated with a factor of 4.72 to 11.62 (Fig. 1A). Similarly, supplementing a *P*. *gingivalis* culture with dead *P*. *intermedia* resulted in a significant 6.02 +/− 3.17 fold increase in *prtC* gene expression and a 2.94 +/− 1.46 fold increase in *fimA* expression (Fig. 1B).

Additionally, the effect of supplementing a *P*. *gingivalis* culture with living *P*. *intermedia* was compared. Although there were no significant differences found in *rgpA*, *rgpB*, *prtC* and *fimA* gene expression between both cultures, the *P*. *gingivalis* culture supplemented with living *P*. *intermedia* showed a 7.29 +/− 4.55 fold higher *serB* and a 53.37 +/− 36.37 fold higher *kgp* expression when compared to the *P*. *gingivalis* culture supplemented with dead *P*. *intermedia* (Supplementary Table 4).

### Cell viability assay

**Figure 2:**
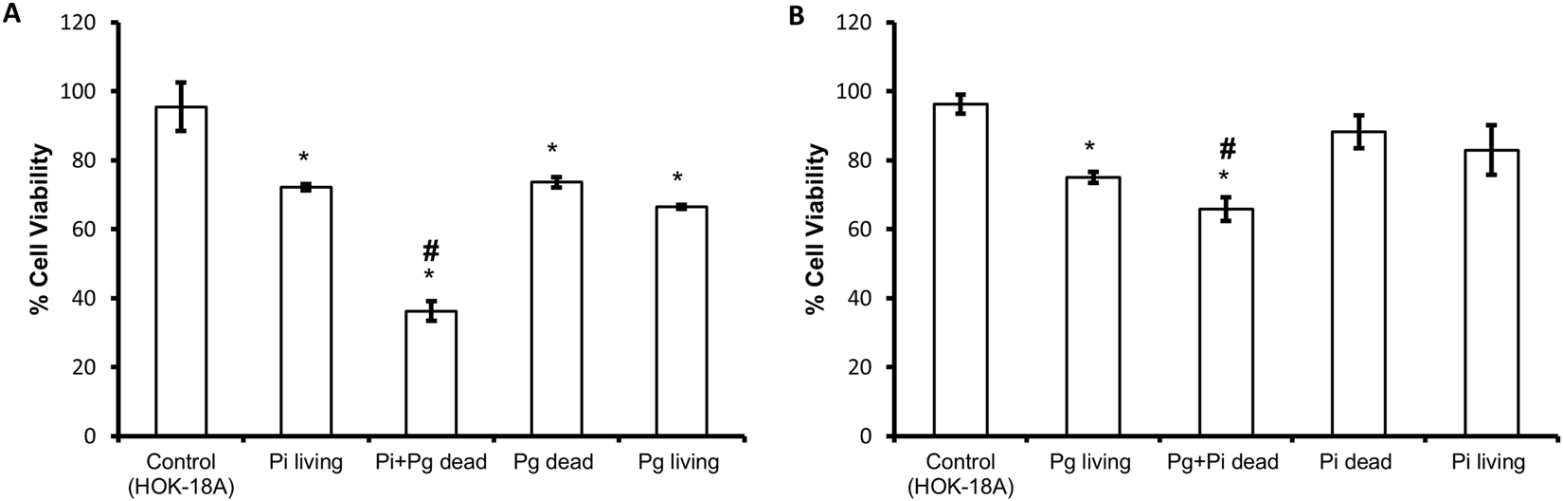
Percentages of cell viability after exposure to dead and living bacteria. (**A**) Percentages of cell viability in presence of living *P*. *intermedia*, dead *P*. *gingivalis*, living *P*. *gingivalis* and the combination living *P*. *intermedia* plus dead *P*. *gingivalis*. (**B**) Percentages of cell viability exposed to living *P*. *gingivalis*, dead *P*. *intermedia*, living *P*. *intermedia* and the combination living *P*. *gingivalis* plus dead *P*. *intermedia*. Control contains only cells without bacteria. *Designates a statistically significant decrease in cell viability respect to control (p < 0.05). ^#^Designates a statistically significant decrease of cell viability respect to all conditions (p < 0.05).

In order to verify whether dead bacteria can reduce cell viability on their own or by increasing *P*. *gingivalis* and *P*. *intermedia* virulence or by the combination of both, a cell viability assay was performed. As shown in Fig. 2, both living and dead *P*. *gingivalis* and *P*. *intermedia* decreased the epithelial cell viability. However, when the epithelial cells were challenged with a combination of living *P*. *intermedia* and dead *P*. *gingivalis*, the cell viability further decreased to 36.30% (±2.83) (p < 0.05) (Fig. 2A). Also when the epithelial cells were challenged with a combination of living *P*. *gingivalis* and dead *P*. *intermedia*, the percentage of cell viability further decreased significantly to 65.87% (±3.44) (Fig. 2B).

## Discussion

The data of this study shows that some oral pathogens can use dead bacteria as a supporting substrate in order to increase their growth in planktonic and biofilm conditions. Such a feature has been named necrotrophy^11^. Although this behaviour is often mentioned, it has never been shown for oral pathogens. Additionally, we show for the first time that in the presence of dead bacteria, oral pathogens increase their virulence, which we name “necrovirulence”. Both aspects can be of importance since the use of toothpastes and mouth rinses, containing bactericidal agents, is a common daily practice in society. It is very well known that these antimicrobials only kill the bacteria in the superficial layers of biofilms^7^. The fate of these bacterial casualties after an antiseptic attack is however not known although these layers of dead bacteria disappear within hours^15^. Therefore it can be hypothesized that these high and dense amounts of dead bacteria could provide multiple nutritional sources, such as C, N, P, Fe, for the surviving bacteria in the same ecosystem. Such behaviour has been shown for *B*. *subtilis* and *L*. *pneumophila* ^11, 16, 17^. Starving *B*. *subtilis* can produce toxins to kill siblings for nutrition (“cannibalism”) and prolong its survival^14^. *L*. *pneumophila*, on the other hand, can use heath killed dead bacteria as a nutritional source to persist in water piping^11^.

The necrotrophic behaviour of the tested oral pathogens showed a dose and species dependency. When exposed to different concentrations of dead bacteria, a clear dose dependent effect was observed on the growth of *P*. *intermedia* and *P*. *gingivalis*. Cultures of *P*. *intermedia* and *P*. *gingivalis* showed a significant increase in growth when there were at least 10 dead cells available to 1 living cell. The necrotrophic behaviour of these pathogens resulted in a supplemental growth of up to 1.46 Log_10_ CFU/mL This magnitude and the dose dependency are similar to what was reported for *L*. *pneumophila* supplemented with dead *Pseudomonas putida* ^11^. The species specificity of the necrotrophy was shown both for the living species as well as for the dead species. In homospecific necrotrophy experiments, the growth increase for *P*. *intermedia* was always stronger than for *P*. *gingivalis*. *F*. *nucleatum* only showed a weak growth increase. These data indicate that the necrotrophic activity specifically depends on the bacterial species. In heterospecific necrotrophy experiments, different dead bacterial species increased the growth of *P*. *intermedia* in different magnitudes, depending on the used dead bacterial species. These data indicate a clear dead species preference. A similar species preference has been shown for *L*. *pneumophila*. Whereas *P*. *putida*, *Escherichia coli*, *Saccharomyces boulardii* and *Acanthamoeba castellanii* increased the necrotrophic growth of *L pneumophila*, *L*. *plantarum* and *B*. *subtilis* did not^11^. Our data showed that the growth of the Gram negative *P*. *intermedia* was much less stimulated by the dead Gram positive *S*. *oralis* and *S*. *gordonii* than by more closely related Gram negative dead *P*. *gingivalis* or *P*. *intermedia*. This could be explained by the robust cell wall structure of these organisms, which prevents nutrients from becoming readily available, as suggested by Temmerman and coworkers^11^.

In order to verify that the dead bacteria were used as a nutritional source, DNA degradation of the dead bacteria was followed by qPCR as a proxy. This technique showed a decrease in dead bacteria over time concomitant to an increase in living bacteria. Such decrease in dead bacteria was not seen in control experiments without living bacteria. These data suggest that the living bacteria were actively breaking down the dead bacteria. However when the bacterial growth medium (BHI) was diluted, the addition of dead bacteria was insufficient to increase the growth of living bacteria, although their survival was improved. It has already been described that a concentration of 10^9^ CFU/mL of dead bacteria is approximately 1 mg/mL of carbon biomass^18^. However, bacterial growth medium (BHI) contains about 30 mg/mL of carbon. In terms of nutritional values, the dead bacteria represent only a low proportion of 30:1 of carbon sources compared to the bacterial growth medium (BHI). Although the dead bacteria might provide other essential nutritional sources, their effect on growth cannot be explained only by nutritional aspects. It can be hypothesized that the presence of dead bacteria can also induce a change of the bacterial phenotype in terms of growth and behaviour.

Since a change in phenotype could be induced by the presence of dead bacteria, the virulence profiles of *P*. *intermedia* and *P*. *gingivalis* were investigated at the gene expression level. Although it is well known that associations of oral pathogenic bacteria can increase their virulence, for instance *P*. *gingivalis* can enhance the attachment and invasion of *T*. *forsythia* to epithelial cells^19^, the effect of dead bacteria on bacterial virulence gene expression has never been investigated. Several well-known virulence genes of *P*. *gingivalis* and *P*. *intermedia* were selected. The data showed that these genes become up-regulated in the presence of dead bacteria. This up-regulation can be explained by the presence of certain specific molecules (inducers) in the solutions of dead bacteria. The gingipain genes *rgpA*, *rgpB*, *kgp* of *P*. *gingivalis* were up-regulated in the presence of dead *P*. *intermedia*. They are involved in several bacterial functions which contribute to survival and virulence. The gingipain genes of *P*. *gingivalis* play an important role in the acquisition of nutrients by degrading peptides from the host or the environment^20, 21^. Their increased expression can therefore be directly related to the observed necrotrophic behavior of *P*. *gingivalis*. Additionally they are also involved in the adhesion, invasion and degradation of host tissues. These genes can also modulate the host immune response by inhibiting IL-2 accumulation in T-cells, by modulating T-cell communication and proliferation^22^. The long fimbriae gene *fimA* was also up-regulated. It encodes for the fimbriae structure, necessary for adhesion to and invasion of host tissues^23, 24^. The up-regulation of *fimA* can be due to the increase in *rgp* gingipains, which process the precursor proteins of long fimbriae^25^. An up-regulation of the collagenase *prtC* gene was also observed. *prtC* has a role in collagen and nutritional peptide degradation^26, 27^. In essence, the virulence genes of *P*. *gingivalis* which were upregulated by dead *P*. *intermedia* all related to the protein metabolism and to adhesion, invasion and destruction of host tissues. On the other hand, dead *P*. *gingivalis* upregulated in *P*. *intermedia* the virulence related genes *kpsD*, *ecf*, *phg*, *inpA* and *adpC*. The polysialic acid transport protein gene *kpsD* and the RNA polymerase ECF-type sigma factor *ecf* gene are involved in the production of a polysaccharide structure which can increase the resistance of *P*. *intermedia* against the host defense^28^. The expression of the hemagglutinin gene *phg* and the cysteine protease interpainA gene (*inpA*) were also increased. The *pgh* gene is involved in the hemolytic degradation of red blood cell to obtain hemoglobin and haem as an exogenous iron source for nutrition^29^. The *inpA* gene is also involved in the haem acquisition by breaking down hemoglobin^30^. It can be hypothesized that these genes are up-regulated in *P*. *intermedia* to acquire the iron compounds from inside of the dead *P*. *gingivalis* cells. Similar to the upregulated invasion genes in *P*. *gingivalis*, the invasin gene *adpC*, involved in the cell invasion of *P*. *intermedia* ^31^, was also up-regulated. A group of heat shock proteins (htpG, dnaJ, dnaK, groeL, groeS, clpB) were also up-regulated in the presence of dead *P*. *gingivalis*. These proteins quickly up-regulate their expression under stressful conditions and can protect the bacterial proteins against external aggression^32^. In essence, dead *P*. *gingivalis* activated genes in *P*. *intermedia* which related to nutrition, protection and invasion of host tissues.

In order to verify whether dead bacteria can reduce eukaryotic cell viability on their own or by increasing *P*. *gingivalis* and *P*. *intermedia* virulence or by the combination of both, a cell viability assay was performed. It is well known that living periodontal pathogens can invade and destroy host tissues^33, 34^. It also described that bacterial compounds like lipopolysaccharide (LPS), bacterial DNA and certain bacterial proteins can induce an inflammatory reaction^35, 36^. However, the influence of dead bacteria (necrotoxicity) alone or in combination with living bacteria on the host tissues has never been shown. Our results showed a lower cell viability for cells challenged with living pathogenic bacteria together with dead pathogenic bacteria when compared to living pathogenic bacteria or dead pathogenic bacteria alone. The increase in cell death can be explained by the increased expression of genes involved in the adhesion, invasion and destruction of host tissues like *rgpA*, *rgpB*, *kgp*, *fimA* and *prtC* in *P*. *gingivalis* and *adpC* in *P*. *intermedia*. However, since dead bacteria alone also reduced cell viability, a combined effect of increase of virulence factors together with toxic compounds of dead bacteria like LPS, bacterial proteins and free bacterial DNA should be considered.

In conclusion, the presence of dead bacteria can stimulate the growth of several oral pathogens (necrotrophy), inducing a new phenotype characterized by an up-regulation of bacterial virulence genes (necrovirulence) and an increased cytotoxicity towards host tissues (necrotoxicity). Such behaviour can also be expected from other human pathogenic species present in other microbiological ecologies such as the gut, the skin and the reproductive tract. These data should warn us for the indiscriminant use of broad-spectrum antimicrobials in conditions where there is an unspecific and incomplete eradication of the whole bacterial population. Therefore, the clinical impact of these results should be investigated.

## Methods

### Bacterial strains and media

*Prevotella intermedia* ATCC 25611, *Porphyromonas gingivalis* ATCC 33277, *Fusobacterium nucleatum* DSM 20482, *Streptococcus gordonii* ATCC 49818, *Streptococcus oralis* DSM 20627, *Streptococcus sanguinis* LM14657, *Streptoccocus mitis* DSM 12643, *Streptococcus salivarius* TOVE-R, *Streptococcus mutans* ATCC 20523, *Streptococcus sobrinus* ATCC 20742, *Actinomyces viscosus* DSM 43327, *Actinomyces naeslundii* ATCC 51655, *Aggregatibacter actinomycetemcomitans* ATCC 43718 and *Veillonella parvula* DSM 2008 were maintained on blood agar (Blood agar Base I, Oxoid, Basingstoke, UK) supplemented with hemin (5.0 mg/mL), menadione (1.0 mg/mL) and 5% sterile horse blood. Broth cultures were prepared in Brain Hearth Infusion (BHI) broth (Difco, Sparks, MD). Multi-species biofilm experiments were performed in Brain Hearth Infusion 2 (BHI-2) broth containing BHI supplemented with 2.5 g/L mucin (Sigma-Aldrich, St. Louis, USA), 1.0 g/L yeast extract (Oxoid, Basingstoke, UK), 0.1 g/L cysteine (Calbiochem, San Diego, USA), 2.0 g/L sodium bicarbonate and 0.25% (v/v) glutamic acid (Sigma-Aldrich, St Louis, USA).

### Preparation of dead bacteria

*P*. *intermedia*, *P*. *gingivalis*, *F*. *nucleatum*, *S*. *gordonii* and *S*. *oralis* were grown on blood agar for 48 hours. These agar plates were exposed to chloroform (Acros organics, Geel, Belgium) vapors for 30 minutes. After exposure, the cultures were removed from the agar plates by a sterile loop and suspended in 3 mL BHI, BHI-2, saliva or serum. This bacterial solution was transferred to an empty Petri dish and exposed to UV light (253 nm) for 30 minutes. The effectiveness of this killing procedure was verified by plating 50 µl of the killed bacterial solutions and control solutions containing living bacteria on a blood agar plate and incubating them for 10 days under aerobic and anaerobic conditions. Absence of bacterial growth confirmed the effectiveness of the killing procedure. Additionally, the killed bacterial solutions and control solutions were subjected to conventional and vitality RT-qPCR. The absence of amplification, when using vitality qPCR on the killed bacterial solutions, additionally confirmed the absence of living bacteria (data not shown). The bacterial concentration was determined by spectrophotometer (OD600, GeneQuant Spectrophotometer, Buckinghamshire, UK) and stock solutions were frozen at −80 °C upon use.

### Necrotrophic growth experiments

3 mL of an overnight culture containing 5 × 10^7^/mL *P*. *intermedia* was supplemented with 3 mL of dead bacteria at 10^9^, 10^8^, and 10^7^ CFU/mL for 24 hours. A culture without dead bacteria (3 mL fresh BHI-2) was used as a control. After 24 hours of anaerobic growth, the cultures were analyzed by plate counting and qPCR.

For the plating experiments, 100 µL from the different conditions were taken and diluted from 10^−1^ until 10^−6^ in physiological water containing 9 g/L of sodium chloride. These dilutions were inoculated on blood agar plates and incubated for 48 hours under anaerobic conditions. Afterwards, *P*. *intermedia* colonies were counted to calculate the final number of bacteria CFU/mL in each condition. The same setup was used to test other bacterial combinations.

### Quantification of bacteria in broth by qPCR

A conventional qPCR technique was used to quantify the total number of *P*. *intermedia* and DNA of dead *P*. *intermedia*, *P*. *gingivalis*, *F*. *nucleatum and S*. *oralis*. A vitality RT-qPCR was also employed to quantify only the living bacterial species in the planktonic and biofilm experiments using specific primers^37, 38^. Both were performed with a CFX96 Real-Time System (Bio-Rad, Temse, Belgium). The Taqman 5’ nuclease assay PCR method was used for detection and quantification of bacterial DNA (already developed for the species). Quantification was based on a plasmid standard curve.

### Necrotrophic growth in multispecies communities

An overnight culture of a bioreactor derived complex multi-species co-culture of 14 species was centrifuged (1438 × g, 10 minutes) and re-suspended in BHI-2, serum or saliva (1 × 10^8^ CFU/mL). 1 mL of this bacterial multi-species solution was inoculated together with 500 µl of 1 × 10^9^ CFU/mL dead or living bacteria in BHI-2, saliva or serum. After 24 hours of anaerobic incubation, 1 mL was taken from each well and analysed via vitality qPCR or/and conventional qPCR. Afterwards, the remaining supernatant was removed and the biofilms at the bottom of the wells were washed with phosphate buffered saline (PBS). The biofilms were detached with 500 µL 0,05% Trypsin-EDTA (Gibco, Paisley, UK) for 15 minutes at 37 °C, transferred to Eppendorf tubes, centrifuged (6010 × g, 10 minutes) and after discarding the trypsin, the biofilm pellets were re-suspended in 1 mL of PBS and analysed by conventional and vitality qPCR.

### Saliva and serum preparation

Unstimulated saliva was obtained for 1 hour per day from a single male volunteer during several days at least 1.5 hours after eating, drinking, or tooth-cleaning. Saliva samples, collected in sterile 50-mL polypropylene tubes, were centrifuged (30 min, 4 °C, 27,000 × g), and the supernatant was pasteurized (60 °C, 30 min) and re-centrifuged in sterile Eppendorf tubes. The resulting supernatants were dispensed into sterile 1.5 mL Eppendorf tubes and stored at −20 °C. The efficacy of pasteurization was tested by plating processed saliva samples onto blood agar (Blood agar Base I, Oxoid, Basingstoke, UK) supplemented with hemin (5 mg/mL), menadione (1 mg/mL) and 5% sterile horse blood. After 72 hours at 37 °C, no CFUs were observed on either aerobically or anaerobically incubated plates. Human serum was obtained by venepuncture of a single, systemically healthy, male volunteer with no oral disease and who had not taken any antibiotics for 1 year. Peripheral venous blood was immediately centrifuged at 264 × g for 30 min at room temperature. The serum was removed and frozen at −20 °C after aliquotation.

### Ethics Statement

The use of human serum and saliva was approved by the ethical committee of the KU Leuven and registered with identifier B322201628215. The procedures were executed according to the Helsinki Declaration and the regulations of the University Hospital, which are approved by the ethical committee. The adult subject provided a written and oral consent after having explained to him the purpose of the study. The subject is aware that the results will be used in a scientific study.

### Bioreactor derived multi-species community

A multi-species community was established in a BIOSTAT B TWIN (Sartorius, Germany) bioreactor. 750 mL of BHI-2 broth was added to the vessel together with 5.0 mg/mL hemin, 1.0 mg/mL menadione and 200 µl/L Antifoam Y-30 (Sigma, St. Louis, USA). The medium was pre-reduced over 24 hours at 37 °C by bubbling 100% N_2_ and 5% CO_2_ in the medium under continuous stirring at 300 rpm. pH was set at 6.7 +/−0.1. After 24 hours, overnight cultures of *S*. *sanguinis*, *S*. *gordonii*, *S*. *salivarius*, *S*. *mitis*, *S*. *oralis*, *S*. *mutans*, *S*. *sobrinus*, *A*. *viscosus*, *A*. *naeslundii*, *P*. *intermedia*, *P*. *gingivalis*, *F*. *nucleatum*, *A*. *actinomycetemcomitans* and *V*. *parvula* were adjusted to an OD of 1.4 and added to the bioreactor. During the first 48 hours, the medium was not replaced. After that, the medium was replaced at a rate of 200 mL/24 hours.

### Virulence gene expression

RNA samples were extracted from the combinations living periodontal pathogen supplemented with 10^9^ dead bacteria and from the periodontal pathogen without dead bacteria after 24 hours using RNeasy Mini Kit (Qiagen, Hilden, Germany). RNA quality was analyzed by nanodrop (Thermo Fisher Scientific, Doornveld, Belgium) and the samples were adjusted to the same concentration before transforming RNA to cDNA through PrimeScript 1^st^ strand cDNA Synthesis Kit (Takara, Shiga, Japan). The expression of virulence genes in the periodontal pathogens was analyzed by qPCR in respect to the house-keeping genes. The primers were extracted from previous publications^31, 37, 39, 40^ or designed based on the genome of *P*. *intermedia* and *P*. *gingivalis* available in the National Center for Biotechnology Information (http://www.ncbi.nlm.nih.gov/) (Table 4).

**Table 4:** Primers of virulence and house-keeping genes from *P*. *gingivalis* (Pg) and *P*. *intermedia* (Pi).

Genes	Sequence	Length
16 S rRNA (Pi) house-keeping	F: CGGTCTGTTAAGCGTGTTGTG R: CACCATGAATTCCGCATACG	99 pb^37^
Hemagglutinin gene *phg* (Pi)	F: TTGCAAGTATTGGTTCGGC R: TCAGGCTGTAAGCGTAGACG	438 pb gb|AF017417.1|:154-1083 GenBank: AF017417.1 protein_id = “AAB70257.1”
Heat shock protein 90 *htpG* (Pi)	F: TGAACGTAAGCCGCAGTTAC R: TTGTTCTTGGCGCAAAGCAG	860 pb gi|965694490:724387-726444 GenBank: AP014598.1 protein_id = “BAU19169.1”
Chaperone *dnaK* gene (Pi)	F: ACGGTGTTGAGGTTGTTGGT R: CTCCGCTGACACTCGGTATC	598 pb gi|965692493:1150302-1152203 GenBank: AP014597.1 protein_id = “BAU17563.1”
Chaperone *dnaJ* gen (Pi)	F: CGAACATTGTCACGGAACAG R: GCAGGAAGTCCTTTGCCTCT	519 pb gi|965692493:412565-413722 GenBank: AP014597.1 protein_id = “BAU16891.1”
Chaperone *groeL* gen (Pi)	F: TGAAGATTTGCGCTGTCAAG R: TAGCTGGAGTTTCTTCAGGT	780 pb gi|965692493:260345-261970 GenBank: AP014597.1 protein_id = “BAU16749.1”
Chaperone *groeS* gene (Pi)	F: AGCGGAGCAGAAAGTAGGTG R: CAACAGCAAGAACGTCGCTT	215 pb gi|965692493:259953-260222 GenBank: AP014597.1 protein_id = “BAU16748.1”
Chaperone *clpB* gene (Pi)	F: CGCTCCATACGAAGCTTTGC R: ATTATCCGTGGCGACGTACC	548 pb gi|965692493:10024-12612 GenBank: AP014597.1 protein_id = “BAU16518.1”
Polysialic acid transport *kpsD* gene (Pi)	F: CGGCAGCTTTAAGTTGTGCC R: GGGTATGACCGATACGCAGG	422 pb gi|877799833:1064644-1065294 GenBank: AP014925.1 protein_id = “BAR95643.1”
RNA polymerase ECF-type sigma factor *ecf* gene (Pi)	F: ATGGAAGCCTCGCAATTCAA R: TCCGTAAGTCCTGTTTCGGC	428 pb gi|965692493:1634435-1634935 GenBank: AP014597.1 protein_id = “BAU17984.1”
Interpain A *inpA* gene (Pi)	F: GAAGGACAACTACAGCGGAAA R: TCCTTTCGTTAGTTCGCTGA	250 pb^39^
Invasin *adpC* gene (Pi)	F: CTGTGTTCTTCAGTTGCACGC R: TATTGCGCCCGCCTTCACCTC	900 pb^31^
16 S rRNA (Pg) house-keeping	F: GCGCTCAACGTTCAGCC R: CACGAATTCCGCCTGC	68 pb^40^
Collagenase *prtC* gene (Pg)	F: TTCTTTGTAGCAGCGGCAGA R: CGTATGCCAACGAGATCGGA	506 pb gi|333802964:798156-799409GenBank: AP012203.1 protein_id = “BAK24920.1”
Arginine-specific cysteine proteinase*rgpA* gen (Pg)	F: CACAACCTTGGCTTCGTTGG R: CAATGGCGCCAAACCTCAAA	508 pb gi|333802964:315477-320528 GenBank: AP012203.1 protein_id = “BAK24470.1”
Arginine-specific cysteine proteinase *rgpB* (Pg)	F: GGAGGAATCTCGTTGGCCAA R: TGCCGACCATATCACCATCG	571 pb gi|333802964:1685747-1687957 GenBank: AP012203.1 protein_id = “BAK25772.1”
Lysine specific cysteine protease *kgp* (Pg)	F: TGGCTTTGGTTGGTGACACT R: AGCCCCTTTCTCCTTAACGC	581 pb gb|AF017059.1|:738-5936 GenBank: AF017059.1 protein_id = “AAC26523.1”
FimA type II fimbrilin *fimA* gen (Pg)	F: TTGCAGGGGTATAAGCACCG R: TTGGAGTTGGCGATGACGAA	567 pb gi|333802964:1331121-1332290 GenBank: AP012203.1 protein_id = “BAK25445.1”

### Cell culture of human oral keratinocytes

Human oral keratinocyte cell line (HOK-18A)^38^ was grown in tissue culture flasks with keratinocyte growth medium (KSFM) supplemented as indicated by the manufacturer (Gibco, Life Technologies Ltd., Paisley, Scotland). Cell culture media were refreshed 3 times a week. After trypsinization, cells were maintained in culture bottles at 37 °C humidified atmosphere containing 5% CO_2_.

### Cell viability assay

Cell viability was evaluated using the XTT cell proliferation assay. XTT powder (Sigma-Aldrich, Diegem, Belgium) was dissolved in RPMI 1640 medium (Life Technologies, Gent, Belgium) at a concentration of 1 mg/mL. Phenazine methyl sulphate (PMS) powder (AppliChem, Darmstadt, Germany) was also dissolved in PBS with a concentration of 0.383 mg/mL PMS. A mixture 50 µl XTT together with 1 µl PMS was added to each well. In brief, HOK-18A^41^ were seeded in 96-well plates (Grenier Bio-One, Frickenhausen, Germany) at a density of 2 × 10^4^ cells per well. The cells were exposed to 5 × 10^7^ CFU/mL of *P*. *intermedia*, 5 × 10^7^ CFU/mL *P*. *intermedia* together with 10^8^ CFU/mL of dead *P*. *gingivalis*, 10^8^ CFU/mL dead of *P*. *gingivalis* and 10^8^ CFU/mL living *P*. *gingivalis* for 20 h at 37 °C. The same combinations were used for *P*. *gingivalis* except for the concentrations of dead and living *P*. *intermedia* which were 10^7^ CFU/mL because dead *P*. *intermedia* killed the cells in higher concentrations. After being exposed, 50 µl of XTT solution was added on the cells in each well for 4 hours. Afterwards, the formazan released by HOK-18A was measured at 450 nm using a micro-plate reader (Multiskan, Thermo Electron Corporation, Vanta, Finland). Cell viabilities are expressed as the percentages in respect to the control without treatment.

### Statistical analysis

All experiments were repeated on 3 different days. A linear mixed model was fitted with run as random factor and growing condition as fixed factor. When residual analysis by means of a normal quantile plot showed that a Log-transformation was recommended, data were Log_10_-transformed. Comparisons between growing conditions and the control were corrected for simultaneous hypothesis testing according to Dunnett. Data were analysed with S-Plus 8.0 for Linux (Tibco, Palo Alto, CA, USA).

## Electronic supplementary material

Online supplementary information
