# Supplementary material for: Necrotrophic growth of periodontopathogens is a novel virulence factor in oral biofilms
Source: Sci Rep. 2017 Apr 24;7:1107. doi: 10.1038/s41598-017-01239-9 (PMC5430626; doi:10.1038/s41598-017-01239-9)
Supplement: Supplementary file 1 — Online supplementary information [file 41598_2017_1239_MOESM1_ESM.pdf]

Online supplementary information

Necrotrophic growth of periodontopathogens is a novel virulence factor in oral biofilms

Esteban Rodriguez Herrero, Nico Boon, Martine Pauwels, Kristel Bernaerts, Vera Slomka, Marc Quirynen, Wim Teughels.

Supplementary Tables:

Supplementary Table 1. Log (MO/mL) of the different species (mean ± standard deviation, n=3) that form part of the 14 species community in presence of living and dead bacteria in saliva.

	Planktonic				Biofilm			
	**Saliva**	**+dead Pi**	**+Living Pi**	**BHI-2**	**Saliva**	**+dead Pi**	**+Living Pi**	**BHI-2**
Aa	5.42±0.07	4.65±0.14#	4.77±0.10#	7.35±0.05*	5.54±0.03	4.18±0.15#	5.51±0.01	6.59±0.09*
Pi	6.51±0.11	6.84±0.04*	7.22±0.07*	7.24±0.06*	7.82±0.03	8.14±0.04*	9.29±0.16*	7.23±0.11#
Pg	7.53±0.11	7.84±0.02*	7.88±0.05*	8.36±0.01*	7.16±0.09	7.66±0.01*	8.38±0.10*	7.82±0.07*
Fn	7.33±0.03	7.57±0.05*	7.30±0.13	8.86±0.08*	8.34±0.02	8.75±0.08*	8.73±0.13*	8.90±0.04*
A.naeslundii	3.37±0.08	0.00±0.00#	2.83±0.46	3.62±0.18	5.40±0.01	2.90±0.18#	4.65±0.08#	6.63±0.24*
A.viscosus	5.54±0.15	3.07±0.25#	5.28±0.08	7.44±0.03*	7.53±0.10	4.52±0.23#	6.64±0.62	8.19±0.05*
S.mutans	5.16±0.06	5.33±0.12	5.07±0.09	6.18±0.05*	6.75±0.08	6.42±0.11#	6.92±0.03	6.60±0.04
S.sobrinus	5.64±0.05	6.07±0.26	5.65±0.12	7.89±0.04*	6.98±0.09	7.22±0.10	6.92±0.12	8.65±0.11*
S.sanguinis	5.39±0.16	4.66±0.25#	5.13±0.14#	6.70±0.04*	6.94±0.14	6.03±0.28#	6.76±0.06	7.95±0.09*
S.gordonii	7.43±0.08	7.68±0.09*	8.12±0.10*	10.22±0.12*	9.37±0.06	8.90±0.06#	9.25±0.10	10.47±0.10*
S.oralis	5.20±0.16	5.23±0.31	4.23±0.16#	6.80±0.13*	7.31±0.04	7.00±0.13#	6.73±0.11#	7.25±0.10
S.salivarius	3.97±0.05	3.74±0.24	3.73±0.46	5.42±0.08*	4.17±0.27	3.76±0.21	3.65±0.05	5.56±0.07*
V.parvula	8.07±0.10	8.00±0.20	7.65±0.10#	8.23±0.01	9.36±0.02	9.33±0.17	9.46±0.05*	9.52±0.12
S.mitis	4.03±0.05	3.52±0.31	4.01±0.13	5.00±0.12*	4.58±0.06	2.83±0.08#	3.76±0.12#	4.53±0.02

Saliva and BHI-2 condition refers to a 14 species community without the addition of dead bacteria.*designates a statistically significant increase of the bacterial concentration in respect to BHI ($p<0.05$). # designates a statistically significant decrease of the bacterial concentration in respect to BHI ($p<0.05$).

Supplementary Table 2. Log (MO/mL) of the different species (mean ± standard deviation, n=3) that form part of the 14 species community in presence of living and dead bacteria in saliva.

	Planktonic				Biofilm			
	**Saliva**	**+dead Pg**	**+Living Pg**	**BHI-2**	**Saliva**	**+dead Pg**	**+Living Pg**	**BHI-2**
Aa	5.23±0.03	4.98±0.06#	5.17±0.29	7.34±0.17*	5.61±0.01	5.14±0.13#	5.66±0.19	7.32±0.12*
Pi	7.18±0.23	7.89±0.08*	7.30±0.20	8.92±0.06*	8.65±0.04	8.80±0.06	8.38±0.12	8.75±0.16
Pg	7.90±0.07	8.67±0.03*	8.74±0.15*	9.86±0.05*	8.75±0.03	8.91±0.06*	10.35±0.02*	10.24±0.14*
Fn	7.34±0.46	7.95±0.17	7.44±0.06	8.36±0.52	8.09±0.11	8.53±0.11*	8.22±0.14	8.92±0.15*
A.naeslundii	5.36±0.25	5.56±0.31	5.50±0.21	5.61±0.18	6.78±0.45	5.26±0.15#	5.76±0.13#	7.36±0.32
A.viscosus	5.78±0.23	5.23±0.17	6.37±0.10	5.92±0.12	7.50±0.07	7.02±0.10#	7.60±0.11	8.31±0.45
S.mutans	3.58±0.02	3.22±0.16#	3.26±0.34	5.20±0.16*	6.13±0.12	6.06±0.04	5.86±0.08#	7.09±0.10*
S.sobrinus	4.96±0.08	4.68±0.07#	4.23±0.18#	5.99±0.09*	5.71±0.07	5.87±0.05*	5.69±0.05	7.22±0.10*
S.sanguinis	5.09±0.40	4.50±0.29	5.05±0.29	6.53±0.07*	7.45±0.08	7.38±0.11	7.28±0.13	8.12±0.06*
S.gordonii	6.84±0.23	6.42±0.25	6.30±0.40	7.55±0.12*	8.63±0.02	8.06±0.15#	8.45±0.10	9.35±0.08*
S.oralis	5.95±0.16	5.65±0.06	5.34±0.06#	5.74±0.11	6.95±0.08	5.44±0.12#	6.09±0.15#	7.25±0.09*
S.salivarius	4.01±0.37	4.20±0.32	4.77±0.42	2.96±0.20#	4.28±0.82	4.13±0.45	4.50±0.92	3.77±0.50
V.parvula	8.98±0.10	8.50±0.19#	8.33±0.15#	9.80±0.15*	10.16±0.05	10.27±0.13	10.02±0.14	11.02±0.10*
S.mitis	5.50±0.09	4.05±0.61#	5.32±0.15	6.18±0.20*	5.78±0.31	4.18±0.68#	5.37±0.42#	7.07±0.06*

Saliva and BHI-2 condition refers to a 14 species community without the addition of dead bacteria.*designates a statistically significant increase of the bacterial concentration in respect to BHI ($p<0.05$). # designates a statistically significant decrease of the bacterial concentration in respect to BHI ($p<0.05$).

Supplementary Table 3. Log (MO/mL) of the different species (mean ± standard deviation, n=3) that form part of the 14 species community in presence of living and dead bacteria in serum.

	Planktonic						Biofilm					
	**Serum**	**+dead Pi**	**+Living Pi**	**+dead Pg**	**+Living Pg**	**BHI-2**	**Serum**	**+dead Pi**	**+Living Pi**	**+dead Pg**	**+Living Pg**	**BHI-2**
Aa	6.84±0.06	7.87±0.08*	7.35±0.15*	6.81±0.16	6.77±0.03	7.72±0.03*	5.93±0.04	5.59±0.38	7.34±0.26*	6.28±0.13*	5.73±0.15	7.88±0.06*
Pi	4.37±0.07	6.78±0.15*	9.43±0.10*	4.31±0.13	5.10±0.03*	8.32±0.07*	3.57±0.06	4.66±0.25*	9.72±0.22*	4.37±0.15*	4.27±0.17*	9.16±0.10*
Pg	8.15±0.04	8.67±0.07*	8.44±0.09*	8.27±0.15	10.33±0.02*	8.44±0.06*	7.04±0.07	7.07±0.35	8.74±0.19*	7.51±0.15*	9.02±0.16*	8.40±0.06*
Fn	4.11±0.05	4.78±0.08*	6.78±0.38*	5.46±0.15*	4.97±0.15*	9.20±0.58*	5.46±0.06	7.88±0.12*	6.79±0.06*	4.74±1.18	6.63±0.58	9.65±0.09*
A.naeslundii	3.93±0.08	5.39±0.20*	3.95±0.24	3.84±0.36	3.90±0.34	5.52±0.06*	2.93±0.13	2.98±0.28	4.11±0.34*	3.71±0.21*	3.19±0.21	6.45±0.17*
A.viscosus	4.33±0.06	6.46±0.13*	4.82±0.08*	4.19±0.22	4.36±0.18	6.64±0.04*	3.03±0.06	3.50±0.37	4.87±0.51*	3.85±0.26*	3.68±0.09*	8.02±0.09*
S.mutans	7.20±0.08	7.33±0.13	6.95±0.09	7.40±0.82	7.40±0.37	7.43±0.06*	6.18±0.02	6.22±0.24	7.46±0.18*	6.80±0.13*	6.53±0.05*	8.31±0.13*
S.sobrinus	6.22±0.04	6.50±0.08*	5.97±0.15	5.99±0.14	6.08±0.12	6.36±0.06	4.62±0.10	4.73±0.24	6.42±0.17*	5.43±0.17*	5.14±0.04*	7.05±0.13*
S.sanguinis	6.35±0.17	6.59±0.24	6.33±0.18	6.72±0.25	7.00±0.27	7.72±0.06*	5.85±0.09	5.83±0.25	7.13±0.22*	6.43±0.15*	5.69±0.39	7.91±0.19*
S.gordonii	8.49±0.09	8.95±0.06*	8.10±0.08#	8.45±0.47	8.79±0.36	9.52±0.05*	7.43±0.07	7.45±0.28	8.82±0.12*	8.35±0.20*	7.95±0.05*	10.74±0.17*
S.oralis	6.35±0.17	6.59±0.24	6.33±0.18	6.72±0.25	7.00±0.27	7.72±0.06*	5.85±0.09	5.83±0.25	7.13±0.22*	6.43±0.15*	5.69±0.39	7.91±0.19*
S.salivarius	4.00±0.03	5.25±0.24*	3.19±0.24#	3.90±0.24	4.72±0.24*	5.54±0.08*	3.83±0.12	4.27±0.25	4.90±0.18*	4.35±0.12	4.01±0.11	6.50±0.16*
V.parvula	8.40±0.03	8.82±0.07*	8.26±0.13	8.04±0.15#	8.23±0.12	8.89±0.12*	7.44±0.08	7.52±0.30	9.04±0.18*	8.29±0.17*	7.61±0.15	10.37±0.09*
S.mitis	3.55±0.73	2.75±0.12	3.18±0.35	4.18±0.13	4.66±0.09	5.55±0.07*	3.57±0.26	3.32±0.39	4.66±0.16*	3.85±0.16	3.23±0.24	6.64±0.20*

Serum and BHI-2 condition refers to a 14 species community without the addition of dead bacteria.*designates a statistically significant increase of the bacterial concentration in respect to BHI ($p<0.05$). # designates a statistically significant decrease of the bacterial concentration in respect to BHI ($p<0.05$).

Supplementary Table 4. Relative fold change of virulence genes from *P. gingivalis* (Pg) after exposure to dead *P. intermedia* (Pi) and living *P. intermedia* (Pi).

Relative fold change		
**Virulence genes**	**Dead Pi**	**Living Pi**
rgpA	1.61±0.27	2.08±0.67
kgp	1.10±0.65	54.47±29.13
rgpB	1.52±0.33	3.70±3.44
prtC	6.02±3.17	10.35±12.78
fimA	2.94±1.46	18.78±16.01
serB	0.75±0.38	8.04±4.01

Relative fold change of virulence genes from *P. gingivalis* after exposure to dead *P. intermedia* and living *P. intermedia*.
